# Supplementary figures and images for: Identification of Crustacean Female Sex Hormone Receptor Involved in Sexual Differentiation of a Hermaphroditic Shrimp
Source: Biomolecules. 2023 Sep 27;13(10):1456. doi: 10.3390/biom13101456 (PMC10604874; doi:10.3390/biom13101456)

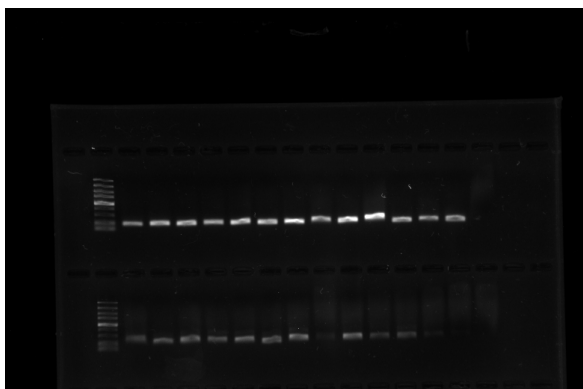

Original WB image of Figure 2A.

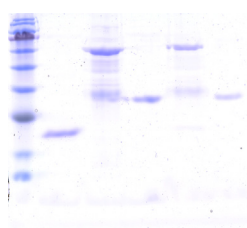

PAGE

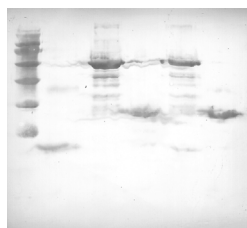

WB

(A)

Original WB images of Figure 3.

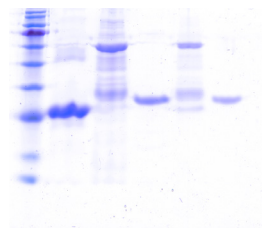

PAGE

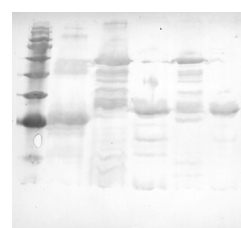

WB

(B)

Supplement: Supplementary file 1 [file biomolecules-13-01456-s001.zip › biomolecules-2595247-supplementary.pdf]
